# Brown Bowel Syndrome in a Middle-Aged Woman with Chronic Idiopathic Malabsorption

**DOI:** 10.1155/2019/4706592

**Published:** 2019-04-04

**Authors:** Renato Parente, Maurizio Pinamonti, Stefania Martina

**Affiliations:** ^1^Department of Pathology, Humanitas-Gradenigo Hospital, Corso Regina Margherita 8, 10153 Torino, Italy; ^2^Department of Surgery, Maria Pia Hospital, Strada Comunale di Mongreno 180, 10132 Torino, Italy

## Abstract

Brown bowel syndrome (BBS) is an extremely unusual condition characterized by an orange-brown discoloration of the bowel and intestinal motility disorders secondary to fat-soluble vitamin deficiency and malabsorption from many different causes. We present the case of a middle-aged woman suffering for years of chronic constipation with recurrent intestinal subocclusion, who was diagnosed BBS on surgical biopsy material. Nutritional supplementary treatment was tried, but her symptoms did not improve, and a decision was finally made in favor of a surgical approach. After subtotal colectomy and continual vitamin nutritional supplementation, she has now regular transit without the use of laxatives. BBS is a rare complication of long-term malabsorption manifesting as intestinal motility disorders, which can lead to severe complications. This condition is only partially responsive to vitamin supplementation, and most cases require surgery.

## 1. Introduction

The so-called “brown bowel syndrome” (BBS) is a rare condition characterized by intestinal motility disorders and an unusual orange-brown pigmentation of tracts or the entire intestine due to the pathologic accumulation of lipofuscin in the cytoplasm of smooth muscle cells [1, 2]. Lipofuscin most probably derives from degeneration of mitochondria, which is caused by a chronic deficiency in fat-soluble vitamins and especially vitamin E [1, 2]. Among the main causes of vitamin deficiency are diseases related to malabsorption, and the most frequently reported are coeliac disease [3–5], severe malnutrition [6], and surgery [7].

We present a case of brown bowel disease in a woman with severe chronic constipation not responsive to laxatives and vitamin nutritional supplementation, who underwent subtotal colectomy with a final diagnosis of BBS.

## 2. Case Report

A 36-year-old woman presented in 2006 at the department of surgery, Maria Pia Hospital, Turin, for important chronic constipation and abdominal pain. She had a long history of constipation with an average of one evacuation every four days despite continual use of laxatives and had been hospitalized several times before for intestinal partial obstruction. Furthermore, the patient suffered of left hemiparesis with difficulty speaking because of a subarachnoid hemorrhage at one year of age. She was implanted a neurostimulator in the third sacral nerve root, but the device was removed two years later due to its inefficacy. In 2010, she was hospitalized again after another partial obstruction, and loop ileostomy was performed. Despite this, the symptomatology did not improve, and the obstructive episodes continued. Colonic manometry and abdominal X-ray revealed a picture of *inertia coli*.

**Figure 1:**
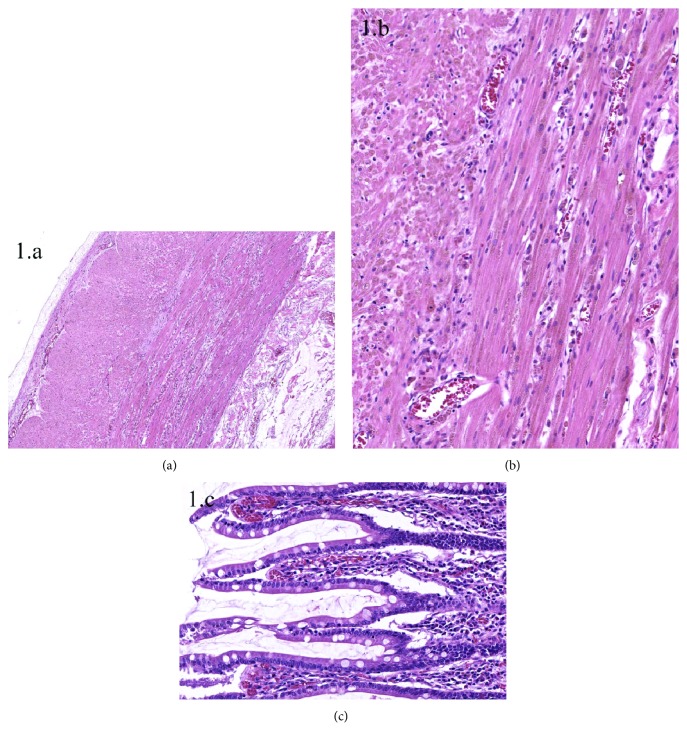
Histological picture of BBS. Small intestinal wall structure appears disrupted at low magnification (a). Closer examination reveals accumulation of brownish granules in the cytoplasm of smooth muscle cells, which occasionally degenerate appearing as single cells between muscle fibers (b). The epithelium, as well as the lamina propria, is normal (c).

On November 2015, during the programmed closing operation of ileostomy, the ileum appeared distended with brownish serosa. Therefore, a decision was taken not to close the ileostomy, and a diagnostic surgical biopsy of the ileum was made. Histologic examination showed an abnormal accumulation of eosinophilic granules in the cytoplasm of smooth muscle cells with disruption of muscular fibers (Figure 1). The mucosa was normal. The pigment was interpreted as lipofuscin, and a suspicion of BBS was raised.

Blood levels of vitamins A, D, E, and K were dosed, and vitamins D and E were found to be low (0.3 mg/dl and 6 ng/ml, respectively). Antibodies against transglutaminase were negative, and there was no clinical or laboratory suspicion of coeliac disease.

**Figure 2:**
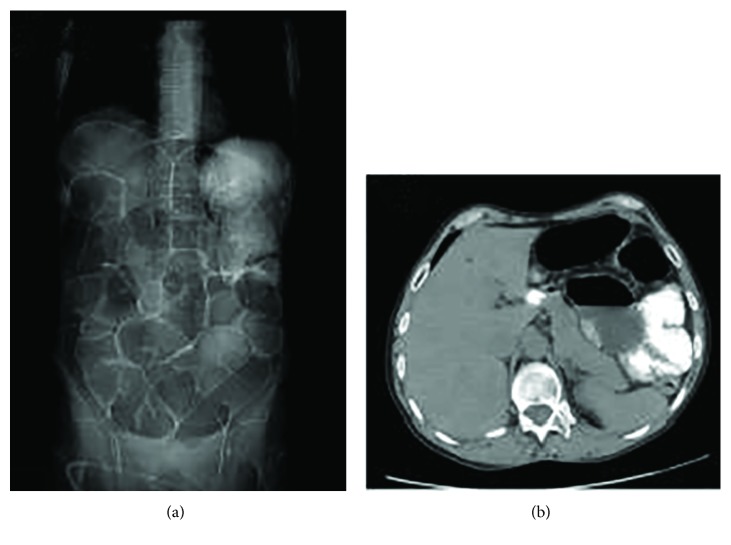
Radiological picture of BBS. Abdominal X-ray (a) and CT scan (b) show massive dilatation of the colon and the small intestine.

After 8 months of nutritional supplementation, the vitamin values were at the lower limit of the normal range (0.8 mg/dl and 10 ng/ml, respectively), but the patient still suffered of recurrent intestinal functional obstruction. Abdominal X-ray and CT evidenced severe intestinal dilatation (Figure 2), indicating the persistence of a severe impairment of colonic motility. Surgery appeared to be the best choice, and after multidisciplinary discussion, on April 2017, the patient underwent subtotal colectomy with maintenance of the rectum as a reservoir. The histological examination confirmed the diagnosis of BBS. The postoperative period was uneventful.

19 months after surgery, the patient is still under vitamin nutritional supplementation, and blood levels of vitamins D and E are still at the lowest acceptable limit, but since then, she did not have any other obstructive episode and has normal daily evacuation without the use of laxatives.

## 3. Discussion

The brown discoloration of the human intestinal wall was first observed by Wagner in 1861 [8]. More than eighty years later, Pappenheimer and Victor studied the accumulation of “ceroid” in various tissues, including the uterine wall, central nervous system, and intestinal musculature, and postulated an origin from dietary lack of vitamin E [9]. The term “brown bowel syndrome” (BBS) was introduced by Toffler et al. in 1963 [1] referring to the orange-brown appearance of the small bowel associated to lipofuscinosis, and since then, this rare pathology has been reported in a limited number of papers in the international literature [3–7, 10–20].

BBS appears to be a consequence of malabsorption of fat-soluble vitamins, especially vitamin E, which has an important protective action against the oxidation of phospholipidic membranes caused by free radicals [7, 14]. Its action is of utmost importance in the mitochondrial walls of smooth muscle cells, where oxidative distress is very high due to the continual metabolic activity. Peroxidation of phospholipids leads to mitochondrial malfunction and degeneration, causing myopathy and intestinal motility disorders. The damaged mitochondria accumulate in the cytoplasm of smooth muscle cells in the muscularis mucosae and muscularis propria of the gastrointestinal tract and can be seen as eosinophilic, PAS-positive granules—lipofuscin—at light microscope examination. This pigmentation may be macroscopically visible as an orange-brown discoloration of the intestinal mucosa and the muscular wall, but sometimes, it is only appreciated at microscopic evaluation. Lipofuscin has been reported not only in the smooth muscle cells of the intestinal wall but also in the apical cytoplasm of enterocytes, in the wall of arteries and veins, in the esophageal wall, in gastrointestinal lymph node macrophages, and even in unusual sites, such as thyroid parenchyma [5, 12].

Coeliac disease is the most frequent condition associated to BBS, followed by chronic pancreatitis, alcohol abuse, Crohn's disease, and other intestinal inflammatory disorders (Table 1). In addition to the symptoms of the underlying disease, such as abdominal pain and diarrhea, patients suffer from the consequences of malabsorption of different nutrients. This condition has a wide spectrum of manifestations, including osseous fragility, defects of coagulation, vulnerability to infections, anemia, and neurologic deficit [17, 21]. The main symptoms of BBS, however, as well as its most severe complications, are related to intestinal motility disorders and include abdominal pain, vomiting, intestinal pseudoobstruction, volvuli, and intussusception. Another symptom that has been reported occasionally is intestinal bleeding, probably due to lipofuscinosis of vascular walls [5]. Complications of BBS might be severe and ultimately fatal [14]. Some authors have postulated a possible association between BBS and malignant tumors, but data are few due to the rarity of the disease, and it is unclear if the carcinogenic effect should be due to vitamin deficiency or other concurrent causes, such as chronic inflammation [4, 19]. An extraintestinal manifestation of the syndrome includes myopathy and neuromuscular dysfunction [7].

BBS might be suspected in the appropriate clinical setting (malabsorption, gastrointestinal motility impairment) when brown pigmentation of the intestinal mucosa is seen at endoscopy. Other conditions associated with brown discoloration of the gastrointestinal tract include Whipple's disease and melanosis coli. A differential diagnosis among these conditions is possible on biopsy material by examining the different natures and localization of the pigment: in BBS, granular pigment is accumulated mainly in the cytoplasm of smooth muscle cells, stains strongly with methenamine silver, Masson-Fontana, and PAS, and is resistant to diastase reaction [7, 16, 22]. Conversely, in both Whipple's disease and melanosis coli, pigment is present almost exclusively in macrophages of the lamina propria [23].

Once the diagnosis is set, patients should be treated with nutritional supplementation in order to correct vitamin deficiency and the other consequences of malabsorption. The intestinal motility disorder should be treated according to its gravity and urgency. Acute and severe complications, such as volvulus and intestinal obstruction, need immediate surgical intervention. In less severe cases, some authors have reported benefits on intestinal motility with regression of the lipofuscin deposits after long-term vitamin E supplementation [16]. Sometimes, however, supportive treatment alone is not capable of restoring intestinal motility and surgical resection of the degenerated intestinal tracts is necessary in order to improve the life quality of patients [7].

## 4. Conclusions

Brown bowel syndrome is a rare complication of long-term malabsorption characterized by lipofuscin deposits and brown discoloration of the intestinal wall. It is caused by vitamin E deficiency and manifests as intestinal motility disorders, which can lead to severe and even fatal complications. Despite the vitamin supplementation treatment, it is only partially responsive to conservative therapy, and most cases require surgery.

## Figures and Tables

**Table 1:** 

Reference	Patient	Underlying disease	Complications
[3]	M 58	Coeliac disease	Massive dilatation of the colon, intestinal bleeding
[3]	F 68	Coeliac disease	-
[3]	F 68	Coeliac disease	Intestinal pseudoobstruction
[5]	M 58	Coeliac disease	Intestinal bleeding
[6]	M 31	Alcohol abuse	Ileal intussusception
[7]	F 58	Jejunoileal bypass (obesity surgery)	Severe osteodystrophy, metabolic complications
[10]	F 67	Crohn's disease	Intestinal obstruction
[12]	M 52	Malabsorption syndrome (unspecified)	-
[13]	M 34	Coeliac disease	Massive dilatation of the colon
[14]	M 52	Endemic sprue	Intestinal pseudoobstruction, volvulus
[16]	F 11	Jejunal atresia at birth (operated in the neonatal period)	Massive dilatation of the small bowel
[16]	M 10	Jejunal atresia at birth (operated in the neonatal period)	Intestinal obstruction
[18]	M 11	Malabsorption syndrome (unspecified)	Massive dilatation of the small bowel
[19]	F 47	Chronic jejunitis	Multiple cancers of the small bowel
[20]	M 79	Malnutrition, alcohol abuse	Massive dilatation of the colon, volvulus
[22]	M 30	Protein loosing enteropathy	Diarrhea
[22]	M 53	Coeliac disease	-
[22]	M 37	Chronic pancreatitis	-
[22]	M 71	Coeliac disease	-
